# Early evolution of radial glial cells in Bilateria

**DOI:** 10.1098/rspb.2017.0743

**Published:** 2017-07-19

**Authors:** Conrad Helm, Anett Karl, Patrick Beckers, Sabrina Kaul-Strehlow, Elke Ulbricht, Ioannis Kourtesis, Heidrun Kuhrt, Harald Hausen, Thomas Bartolomaeus, Andreas Reichenbach, Christoph Bleidorn

**Affiliations:** 1 Sars International Center for Marine Molecular Biology, University of Bergen, 5008 Bergen, Norway; 2 Paul-Flechsig-Institute for Brain Research, University of Leipzig, 04103 Leipzig, Germany; 3 Translational Center for Regenerative Medicine, University of Leipzig, 04103 Leipzig, Germany; 4 Carl-Ludwig-Institute for Physiology, University of Leipzig, 04103 Leipzig, Germany; 5 Institute of Evolutionary Biology and Ecology, University of Bonn, 53121 Bonn, Germany; 6 Department of Integrative Biology, University of Vienna, 1090 Vienna, Austria; 7 Biotechnology Center, Technische Universität Dresden, 01307 Dresden, Germany; 8 Museo Nacional de Ciencias Naturales, Spanish National Research Council (CSIC), 28006 Madrid, Spain

**Keywords:** Deuterostomia, glia, nervous system, Reissner's fibre

## Abstract

Bilaterians usually possess a central nervous system, composed of neurons and supportive cells called glial cells. Whereas neuronal cells are highly comparable in all these animals, glial cells apparently differ, and in deuterostomes, radial glial cells are found. These particular secretory glial cells may represent the archetype of all (macro) glial cells and have not been reported from protostomes so far. This has caused controversial discussions of whether glial cells represent a homologous bilaterian characteristic or whether they (and thus, centralized nervous systems) evolved convergently in the two main clades of bilaterians. By using histology, transmission electron microscopy, immunolabelling and whole-mount *in situ* hybridization, we show here that protostomes also possess radial glia-like cells, which are very likely to be homologous to those of deuterostomes. Moreover, our antibody staining indicates that the secretory character of radial glial cells is maintained throughout their various evolutionary adaptations. This implies an early evolution of radial glial cells in the last common ancestor of Protostomia and Deuterostomia. Furthermore, it suggests that an intraepidermal nervous system—composed of sensory cells, neurons and radial glial cells—was probably the plesiomorphic condition in the bilaterian ancestor.

## 1. Background

The origin and evolution of animal nervous systems is controversially discussed [1–3]. In particular, it still remains an open question whether the complex centralized nervous system (CNS) found in the different clades of bilateral-symmetric animals (Bilateria) evolved independently out of a non-bilaterian nerve-net, or if this highly specialized bilaterian tissue shares a common origin [4–6]. The vast majority of species comprises two main clades within Bilateria: the deuterostomes, including all vertebrates, echinoderms, hemichordates, cephalochordates and tunicates; and the protostomes, including taxa such as annelids, molluscs, insects and nematodes. Most of these taxa have complex and CNS consisting of several neuron types with comparable molecular pathways, function and morphology [7,8]. Whereas most comparative neural investigations focus on different neuronal cell types supposed to be involved into various ways of stimulus perception and signal transmission, another important cell type also being part of bilaterian nervous systems is often neglected in comparative studies: the glial cells.

Glial cells are known to represent a class of non-neuronal supportive cells, constituting a common feature of the CNS in Bilateria. However, it still remains controversial whether glial cells are homologous across deuterostome and protostome animals or evolved convergently in the bilaterian main clades [9]. Although supposed to be involved in almost all neural functions [10,11], these cells seem to be absent in several bilaterian taxa, as well as in non-bilaterian animals [9]. In particular, the epithelial nervous system of the Deuterostomia is occupied by a specific bipolar type of glial cells that span the entire thickness of the epidermis forming a regular ‘scaffold’, which are called radial glia [12]. In chordates, radial glial cells arise from neuroepithelial stem cells in the neural plate early during embryogenesis; they also represent neuronal stem cells that give rise to various types of other glial and neuronal cell types of the CNS [13–15]. Outside the Deuterostomia, however, the presence of radial glial cells has not yet been reported [16].

To examine the presence of radial glia-like cells also outside the Deuterostomia, we investigated taxa representing all major branches of the bilaterian tree and especially focused our study on non-deuterostomian groups possessing a supposedly plesiomorphic intraepidermal nervous system. By using an integrative approach including histology, transmission electron microscopy (TEM), immunolabelling and whole-mount *in situ* hybridization (WMISH), we show here that protostomes also possess radial glia-like cells, which are very likely to be homologous to those of deuterostomes. This implies an early evolution of radial glial cells in the last common ancestor of protostomes and deuterostomes (Nephrozoa). Moreover, our antibody staining suggests that the secretory character of radial glial cells is apparently maintained throughout their various evolutionary adaptations. So far, neuro- and gliogenesis originating from radial glial cells are only described for deuterostomes [13–15,17–19]. However, our results suggest that this cell type takes over important roles in nervous systems throughout Nephrozoa.

## 2. Material and methods

### (a) Collection and fixation of specimens

For details, refer to the electronic supplementary material, table S1. Divergent collection and fixation details are specified where required.

### (b) Larval rearing and culturing of *Owenia fusiformis*

Different larval stages of *Owenia fusiformis* were cultured under laboratory conditions and fixed as described previously [20].

### (c) RNA-Seq and transcriptome assembly

Total RNA was extracted from cryofixed larvae using the Agencourt RNAdvance Tissue Kit (Beckman Coulter, Indianapolis, IN, USA). Library preparation and sequencing was performed by EMBL Genomics Core Facility (Heidelberg, Germany) using cation-based chemical fragmentation of RNA, Illumina Truseq RNA-Sample Preparation Kit and one lane of 100 bp paired-end read sequencing on Illumina HiSeq 2000. Raw reads were trimmed and error corrected with Cutadapt 1.2.1 [21], the ErrorCorrectReads tool implemented in Allpaths-LG [22] and assembled with Trinity [23].

### (d) Gene cloning and RNA probe preparation

Contig sequences for the investigated genes were identified in the transcriptome dataset by bidirectional BLAST [24]. Whole transcripts or fragments were amplified by PCR with specific primers (Fw = AGTTTGGGATGGTGGG, Rv = TTCTGGGCTAGCTGGT) from cDNA prepared with SuperScript III (Invitrogen, Waltham, MA, USA), ligated into pgemT-easy vector (Promega, Madison, WI, USA) and cloned into Top10 chemically competent *Escherichia coli* (Invitrogen). Clone sequences were verified by Sanger sequencing. DIG-labelled sense and antisense RNA probes were generated from plasmid DNA with T7- and SP6-RNA polymerases (Roche, Madison, WI, USA).

### (e) Gene orthology

Reciprocal blast yielded unambiguous results for gene orthology assignment of Ofu-subcommissural organ (SCO). Furthermore, public databases (Genebank, JGI, Uniprot) and the transcriptome were screened for homologues by text search and BLAST with respective query sequences. For comparison with other members of the Thrombospondin-family, protein domains were analysed using SMART [25,26]. An overview is given in electronic supplementary material, figure S2. Accession number: MF358540.

### (f) Immunohistochemistry, confocal laser scanning microscopy and image processing

Whole-mount preparations and vibratome sections were analysed. Specimens were anaesthetized in 7% MgCl_2_ in saltwater. For fixation, refer to electronic supplementary material, table S1. After rinses in 0.1 M phosphate-buffered saline (PBS) for at least 2 h, specimens were stored in PBS containing 0.05% NaN_3_ at 4°C. For vibratome sectioning, specimens were rinsed in 0.1 M PBS and embedded in gelatin/albumin medium. The blocks were cut with a VT1000S vibratome (Leica Microsystems, Wetzlar, Germany) into 80–100 µm thin sections. The sections were washed in PBS containing 0.1% Triton X-100 (PTA). Antibody staining was preceded by tissue permeabilization for 1 h in 0.1 M PBS containing 0.1% NaN_3_ and 0.1% Triton X-100 (PTA), suited by incubation in block-PTA (6% normal goat serum; Sigma-Aldrich, St Louis, MO, USA) overnight. For Hemichordata and Priapulida, PTA contained 2% Triton X-100. The primary antibody polyclonal rabbit antiserum against bovine RS (SCO-K10) (W. W Naumann, University of Leipzig, dilution 1 : 1000) was applied for 24–72 h in block-PTA. Afterwards, specimens were rinsed in block-PTA and incubated with secondary fluorochrome-conjugated antibodies (goat anti-rabbit Alexa Fluor 488, Invitrogen, dilution 1 : 500) in block-PTA for 24–48 h. Subsequently, samples were washed in 0.1 M PBS (without NaN_3_), stained with DAPI for 15–30 min (5 mg ml^−1^ stock solution, final concentration 10 µg ml^−1^) and washed for 2 × 5 min in 0.1 M PBS. For experiments including larval and juvenile stages of *O. fusiformis*, specimens were rinsed for 2 × 5 min in PTW (PBS with 0.1% Tween 20) at room temperature (RT) and transferred into 10 µg proteinase K ml^−1^ PTW for 2–3.5 min depending on the developmental stage (24 hpf–3 dpf = 90 s; 7 dpf = 2 min; 14–21 dpf = 2.5 min; after metamorphosis = 3.5 min). After two short rinses in glycine (2 mg glycine ml^−1^ PTW), and 3 × 5 min washes in PTW, the specimens were refixed using 4% PFA in PBS containing 0.1% Tween for 20 min at RT. Subsequently, the developmental stages were rinsed for 2 × 5 min in PTW and 2 × 5 min in THT (0.1 M Tris–Cl, 0.1% Tween), and blocked for 1–2 h in 5% sheep serum in THT according to the protocol of Conzelmann & Jékely [27]. The primary antibody polyclonal rabbit antiserum against bovine RS (SCO-K10) (W. W Naumann, University of Leipzig, dilution 1 : 1000) was applied for 24–72 h in THT containing 5% sheep serum at 4°C. Specimens were then rinsed for 2 × 10 min in 1 M NaCl in THT and 5 × 30 min in THT, and incubated subsequently with secondary fluorochrome-conjugated antibodies (goat anti-rabbit Alexa Fluor 488, Invitrogen dilution 1 : 500) in THT containing 5% sheep serum for 24 h at 4°C. Subsequently, the samples were washed for 6 × 30 min in THT, stained with DAPI for 10–15 min (5 mg ml^−1^ stock solution, working solution: 2 µl in 1 ml THT—final concentration 10 µg ml^−1^) and washed for 2 × 5 min in THT. Samples were dehydrated in an ascending isopropanol series, treated in Murray's clearing solution (benzyl alcohol plus benzyl benzoate, 1 : 2) and mounted between two coverslips in DPX (Sigma-Aldrich) or embedded in 90% glycerol/10% 10× PBS containing DABCO. Standard negative staining controls were performed for all antibodies, and in all cases, the omission of the primary and/or secondary antibody resulted in no staining. Cross-reactivity analyses and immunoprecipitation for antigen characterization of the used SCO-K10 antibody were performed in earlier studies [28,29]. Samples were analysed with Leica TCS STED and Leica TCS SP5 microscopes (Leica Microsystems, Wetzlar, Germany). Z-stacks were processed with Leica AS AF v. 2.3.5 (Leica Microsystems) and Imaris 8.2 (Bitplane AG, Zurich, Switzerland). Final panels were designed using Adobe (San Jose, USA) Photoshop CC and Illustrator CC.

### (g) Whole-mount *in situ* hybridization

Larvae and juveniles of *O. fusiformis* were fixed for 2.5 h in 4% PFA in phosphate buffer with Tween (PTW; pH 7.4) and stored at −20°C in methanol until usage. The *in situ* hybridization procedure was performed as described previously [30] with some modifications: Proteinase K concentration was reduced to 5 ng ml^−1^, Proteinase K treatment was 3.5 min for late larvae and 5 min for juveniles, hybridization buffer contained 5% dextran sulfate, the incubation time was 72 h and final staining was done with Fast Blue (Sigma-Aldrich, MO, USA). To evaluate staining, significance control experiments with sense probes were made. Furthermore, we combined *in situ* hybridization and antibody staining, by processing specimens after *in situ* hybridization with the immunohistochemistry procedure mentioned above.

### (h) Ultrathin sections and transmission electron microscopy

Embedding was performed using the automated tissue processor (Leica Lynx Processor, Leica Microsystems GmbH, Saarland, Germany). After rinses of 4 × 20 min in 0.1 M PBS at 4°C, samples were treated for 2 × 60 min with 1% osmium tetroxide (Carl Roth, Karlsruhe, Germany) at 4°C. Afterwards, samples were rinsed for 20 min in 0.1 M PBS at 4°C and 20 min at RT. Subsequently, samples were dehydrated using an ascending acetone series at RT. The 70% acetone solution contained 1% phosphotungstic acid (Fluka, Sigma-Aldrich, Munich, Germany) and 1% uranyl acetate (Serva, Heidelberg, Germany) for post-contrasting. The samples were then gradually embedded at RT in non-hardening epoxide resin (Durcupan ACM Fluka; Sigma)-acetone mixtures (1 : 3, 1 : 1, 3 : 1; 60 min each), incubated in pure Durcupan and successively replaced by hardening Durcupan. After polymerization at 60°C for 2 days, semi-thin (500 nm) and ultrathin (60–65 nm) sections were cut with a microtome (Leica Ultracut UCT, Leica Microsystems, Saarland, Germany), employing a diamond knife. All sections were frontally orientated; displayed electron micrographs of *O. fusiformis* derived from the trunk of the worm, of *Balanoglossus misakiensis* from the posterior proboscis and from *Asterias rubens* from the isolated radial nerve.

For electron microscopy, ultrathin sections were transferred onto formvar-resin-laminated slot grids (Plano, Wetzlar, Germany) and post-contrasted with 3% uranyl acetate and 3% lead citrate. Sections were examined with a Sigma-0231 scanning electron microscope (27 kV; Zeiss), employing a STEM detector and ATLAS software (Zeiss).

### (i) Histology and semi-thin sections

*Balanoglossus misakiensis* (Hemichordata) specimens were relaxed in 7% MgCl_2_ in seawater for 5–10 min and fixed in ice-cold 2.5% glutaraldehyde in 0.05 M phosphate buffer+0.3 M sodium chloride (pH 7.4). Primary fixation was stopped after 45 min with three rinses in buffer. Post-fixation with 2% OsO_4_ was carried out for 30 min and stopped with three buffer rinses (15, 30, 30 min) followed by two rinses with ddH_2_O (15, 30 min). After a graded series of ethanol specimens were embedded in Epon resin. Semi-thin sections (0.5 µm) of *B. misakiensis* (3 days post settlement) were carried out using a Leica Ultracut S. Sections were stained with toluidin blue and imaged with an Olympus BX-UCB mounted on an Olympus BX51 compound microscope.

*Owenia fusiformis* and *Asterina gibbosa* (collected in 2009 in Concarneau, France) were relaxed in a 7% MgCl_2_ solution and fixed in Bouin's fluid (modified after Dubosq-Basil) overnight. Adult specimens of *Priapulus caudatus* originated from a stock at the Natural History Museum of Denmark (ZMUC PRI-00119; collected in 1927 in Agparmiut, Greenland) and were fixed in Bouin's fluid. All animals were dehydrated in an ascending ethanol series, incubated in methyl benzoate and butanol, preincubated in Histoplast (Thermo Scientific, Dreieich, Germany) at 60°C for 3 days with medium changes and embedded in Paraplast (McCormick Scientific, Richmond, VA, USA). Sections (5 µm) were made using a Reichert-Jung Autocut 2050 microtome (Leica, Wetzlar) and put on albumen–glycerol-coated glass slides. According to a modified Azan staining method, sections were stained with Carmalaun, differentiated with sodium phosphotungstate (5%), washed in dH_2_O, stained with aniline blue orange G and embedded with Malinol (Waldeck, Münster, Germany). In this staining, nervous system appears grey/reddish, nuclei of neuronal somata red and extracellular matrix blue. Musculature stains orange. Slices were analysed with an Olympus microscope (BX-51) and images taken with an Olympus ccl2 camera equipped with the dot slide system (Olympus, Hamburg). Slices were aligned with imod and imod align (http://www.q-terra.de/biowelt/3drekon/guides/imod_first_aid.pdf).

## 3. Results and discussion

**Figure 1.**
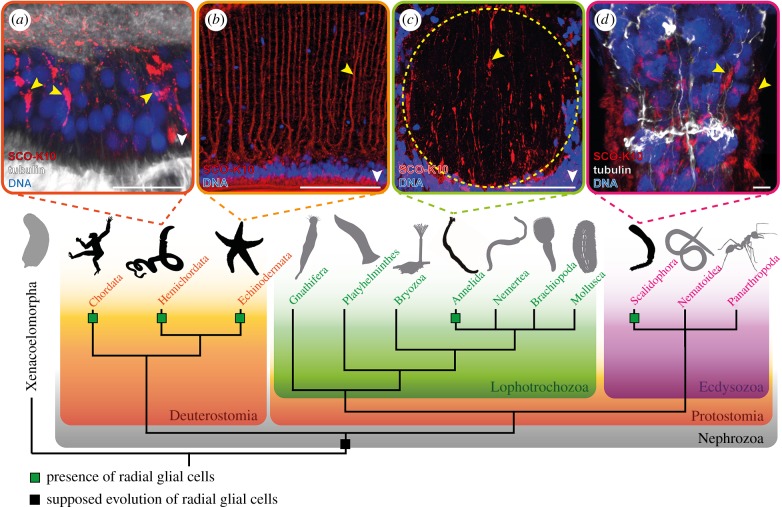
Phylogenetic hypothesis concerning Bilateria based on Cannon *et al*. [37], and SCO-K10-like immunoreactivity (-LIR) for selected taxa. Schematic images for radial glial cell-positive taxa are black, others are grey. Cross-sections of the nervous system of (*a*) *Balanoglossus misakienis*, (*b*) *A. rubens*, (*c*) *O. fusiformis* and (*d*) whole mount of the anterior end of *P. caudatus*; *z*-projections; external epidermal = down and pointed by the white arrowhead (left and right in (*d*)); SCO-K10-LIR reveals the presence of elongated cells (yellow arrowhead) within epidermis. The yellow dotted line marks the position of the nerve cord in (*c*). Scale bars, 20 µm (*a*–*c*); 5 µm (*d*). Schematic images from phylopic.org.

We used histological staining, TEM investigations of ultrathin sections, immunolabelling and WMISH to analyse the intraepidermal nervous system of several taxa. Our antibody staining is based on the immunolabelling of SCO-spondin, using a polyclonal antibody (SCO-K10) derived from bovine Reissner's substance and directed against bovine SCO-spondin [28,29]. This extracellular matrix glycoprotein is known to be expressed and secreted by the vertebrate SCO [31] and deuterostomian radial glial cells in general [32]. SCO-spondin is involved in early cell migration and axonal guidance, and seems to play important roles in vertebrate neurogenesis [33]. For our comparative investigations, we focused on taxa that represent the main branches of Nephrozoa [34] and exhibit an intraepidermal nervous system. In contrast with an intraepidermal plexus, an intraepidermal nervous system consists of one or several nerve cords that locally replace the epidermis [35]. This type of nervous system organization is presumably plesiomorphic for Bilateria [36]. Thus, we investigated the annelid *O. fusiformis* (Protostomia, Lophotrochozoa), the priapulid *P. caudatus* (Protostomia, Ecdysozoa) and the enteropneust *B. misakiensis* (Deuterostomia, Hemichordata) (figure 1). For comparisons, we re-examined the sea star *A. rubens* (Deuterostomia, Echinodermata) (figure 1), where the here-applied antibody had already been tested successfully [29].

**Figure 2.**
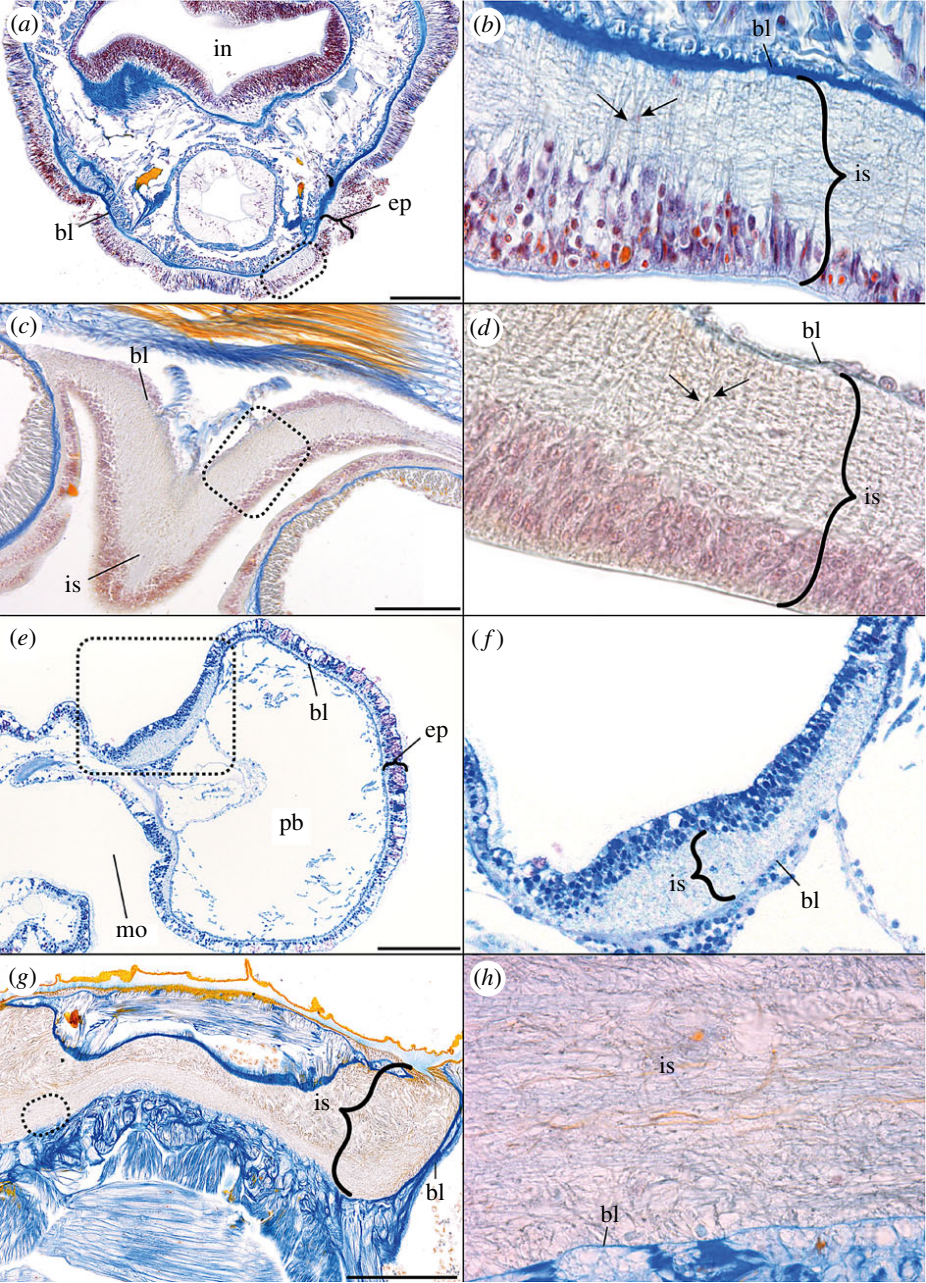
Semi-thin sections of the neuroepithelium in (*a*,*b*) *O. fusiformis*, (*c*,*d*) *A. gibbosa*, (*e*,*f*) *B. misakiensis* and (*g*,*h*) *P. caudatus*. Azan (*a*–*d*,*g*,*h*) and toluidine-blue (*e*,*f*) staining. Azan stains somata and cell processes reddish, neuropil grey, nuclei red and extracellular matrix blue. Musculature is orange. Toluidine-blue stains somata, cell processes and extracellular matrix appear in different blue intensities. (*a*,*c*,*e*) The intraepidermal nervous system (is) locally replaces the epidermis (ep) (bl, basal lamina; in, intestine; mo, mouth; pb, proboscis). (*b*,*d*,*f*,*h*) Higher magnification of regions indicated by dotted line in (*a*), (*c*), (*e*) and (*g*), respectively (no scale bar included). Arrows indicate radial structures in (*b*) and (*d*). Scale bars, 100 µm.

**Figure 3.**
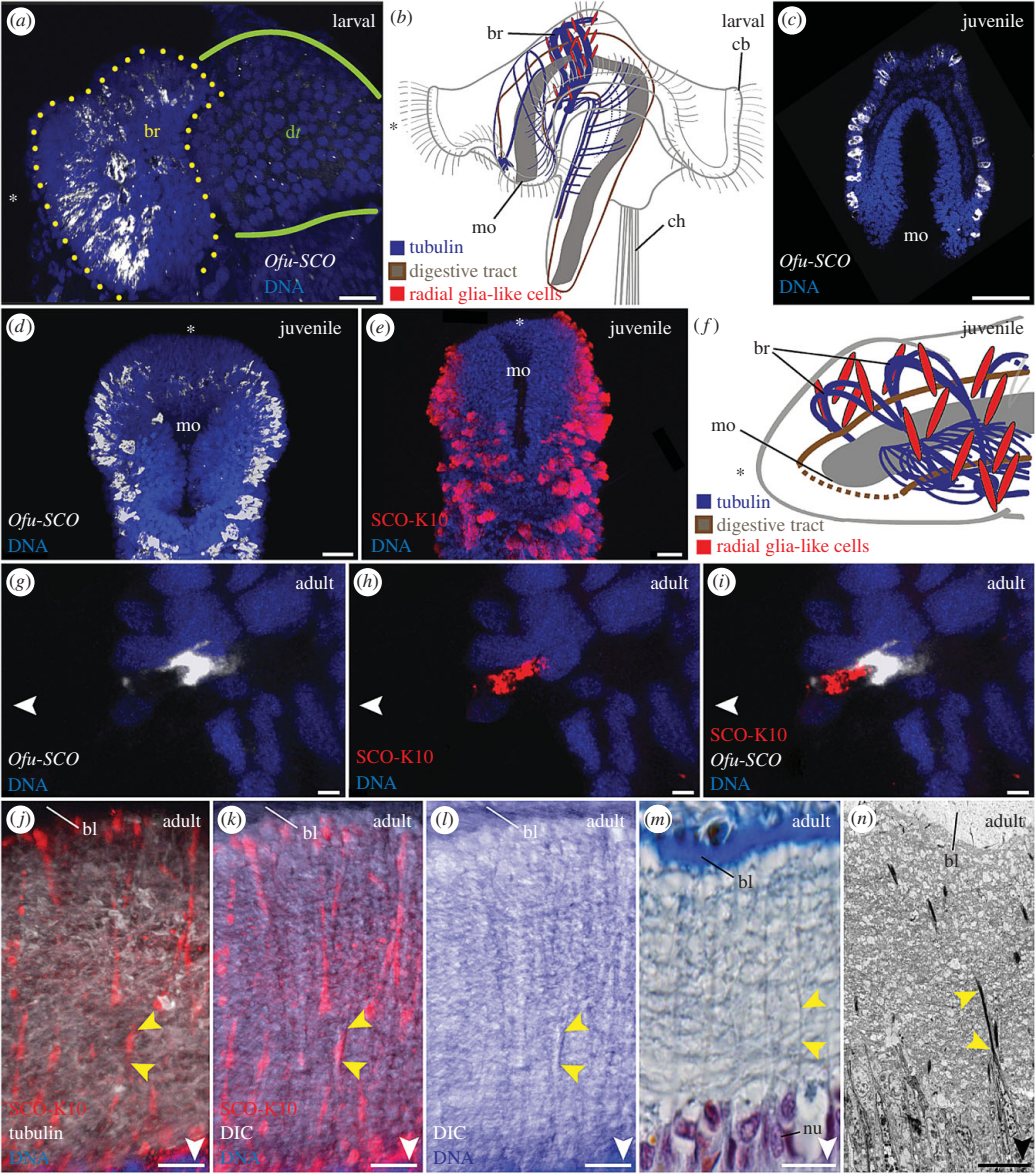
*Ofu-SCO* expression and SCO-K10 antibody staining in different developmental stages of *O. fusiformis*. Confocal *z*-projections (*a*–*c*,*e*,*g*–*l*), Azan staining (*m*), TEM image (*n*) and schematic drawings (*b*,*f*). Anterior is indicated by an asterisk and apical is pointed by the white arrowhead. (*c*,*j*–*n*) represent cross-sections. (*a*,*b*) In late larvae, the *Ofu-SCO* expression is restricted to the larval brain (yellow dotted line). (*b*) The scheme represents a simplified pattern of SCO-positive cells close to the apical larval nervous system. (*c*,*d*) *Ofu-SCO* mRNA is expressed intraepidermally in the anterior end. (*e*) SCO-K10-LIR reveals a congruent pattern. (*f*) The scheme illustrates a simplified pattern of SCO-positive cells close to the anterior nervous system. (*g*–*i*) *Ofu-SCO* expression and antibody staining SCO-K10 reveal a colocalization and spatial separation of both signals. (*j*–*n*) The anterior ventral nerve cord of adult worms exhibits distinct radially arranged cells (yellow arrowheads) that traverse the entire epidermis, show SCO-K10-LIR and possess distinct intermediate filaments. bl, basal lamina; br, brain; cb, ciliary band; ch, chaetae; DIC, differential interference contrast; dt, digestive tract; mo, mouth opening; nu, nucleus. Scale bars, 20 µm (*a*,*c*–*e*), 2 µm (*g*–*i*) and 7 µm (*j*–*n*). Schematic drawings (*d*,*f*) are modified from [20].

**Figure 4.**
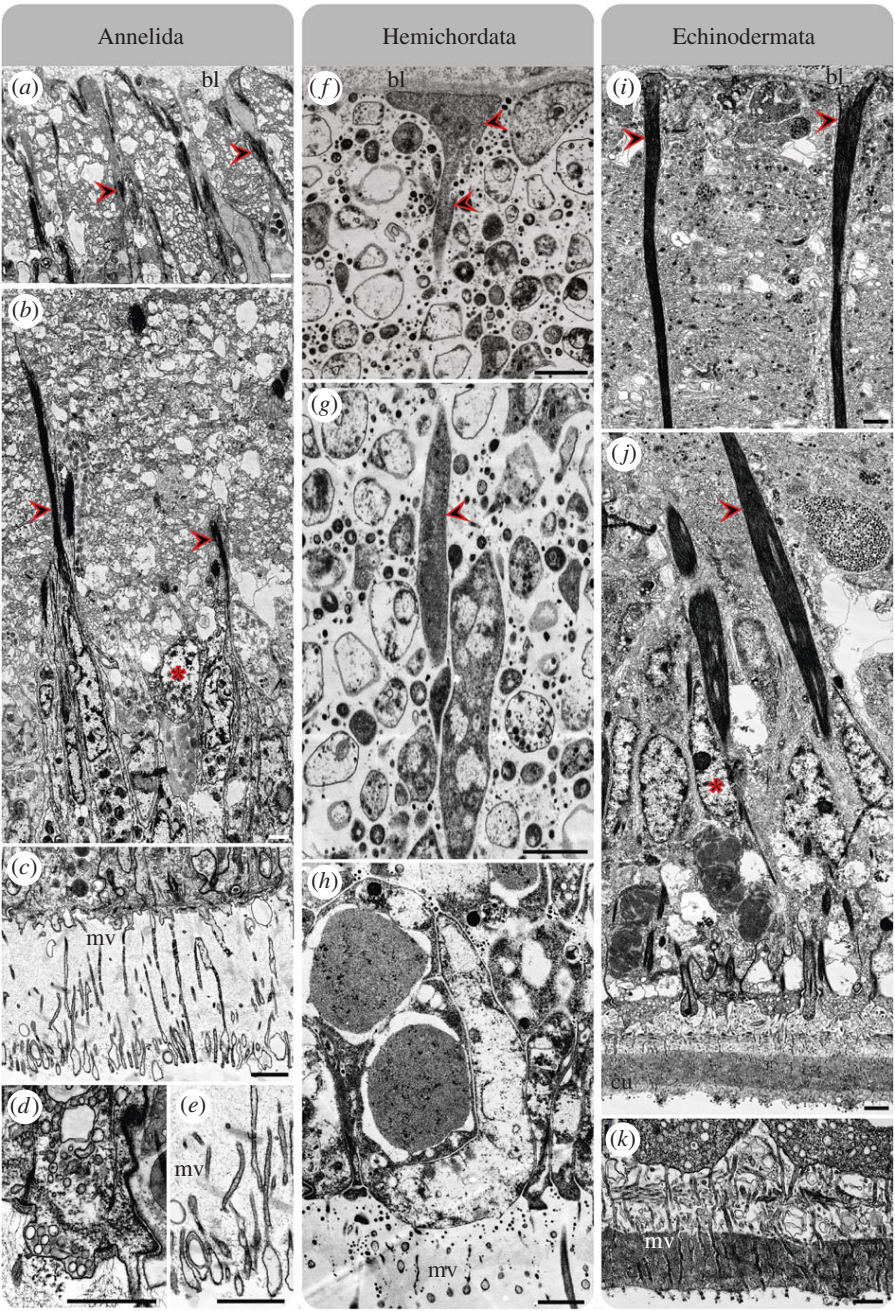
Ultrastructure of glial components in the neuroepithelium of (*a*–*e*) *O. fusiformis*, (*f*–*h*) *B. misakiensis* and (*i*–*k*) *A. rubens*. (*a*,*f*,*i*) Funnel-shaped basal processes (including endfeet) of radial glial cells (arrowheads) terminate at inner epithelial surface (bl, basal lamina). (*b*,*g*,*j*) Inner cell processes of radial glial cells (arrowheads) traverse whole epithelium up to apical nuclear layer; asterisks indicate the location of glial cell somata. (*c*,*e*,*h*,*k*) Microvilli (mv) from radial glia protrude and break through cuticle (cu). (*d*) Presumable radial glial cell during release of secretory products into the cuticle. Scale bars, 1 µm.

**Figure 5.**
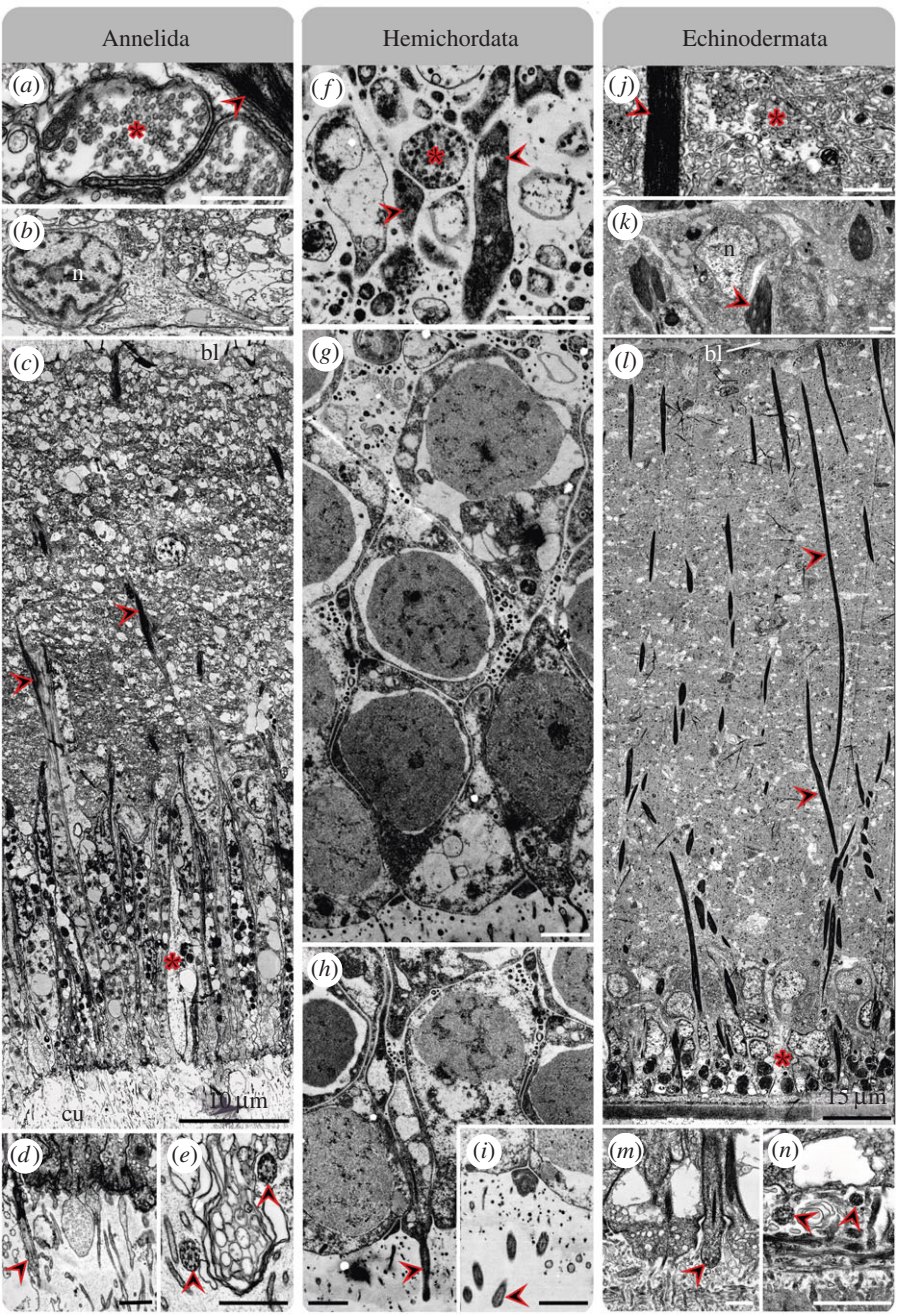
Neuronal components in the neuroepithelium of (*a*–*e*) *O. fusiformis*, (*f*–*i*) *B. misakiensis* and (*j*–*n*) *A. rubens*. (*a*,*f*,*j*) Neuronal cells, which include synaptic vesicles (asterisks), are flanked by radial glial processes (arrowheads). (*b*,*c*,*g*,*k*,*l*) Most of neuronal (*n*, neuronal nucleus) and glial cell somata (asterisk, supposed location) located in apical nuclear layer of epithelium (bl, basal lamina). (*d*,*e*,*h*,*i*,*m*,*n*) Cilia (arrowheads) at the cell surface and in the cuticle (cu). Scale bars, 1 µm and as indicated.

The oweniid *O. fusiformis* is part of the earliest-branching split of annelids [39]. We found that the ventral nerve cord of this species is situated intraepidermally (figure 2*a*,*b*), thereby confirming results of an anatomical study on another oweniid species [40]. A higher magnification reveals the presence of distinct, regularly arranged structures traversing the entire epidermis from the basal lamina towards the epithelial surface. Examination of the same region using an antibody directed against SCO-spondin reveals prominent SCO-K10-like immunoreactivity (-LIR) in these cells (figure 1*a*). In fact, the ventral cord and brain of *O. fusiformis* exhibit radially arranged SCO-K10-LIR and distinct gene expression of *Ofu-SCO-spondin*, crossing epidermis and intraepidermal nervous system (figures 1*c* and 3*a*–*f*,*j*–*n*). Notably, a spatial separation of the SCO-protein in the apical cell area and of the *Ofu-SCO* mRNA in basal parts of the cell is observable (figure 3*g*–*i*). Alignment of immunohistochemical, histological and ultrastructural investigations (figure 3*j*–*n*) confirms the exclusive presence of radially arranged cells traversing the intraepidermal nerve cord and the adjacent epidermis, featuring SCO-K10-LIR, exhibiting prominent intermediate filaments (figures 4*b* and 5*c*) and flanking neuronal cells with synaptic vesicles (figure 5*c*). These cells possess funnel-shaped endfeet terminating at the basal lamina (figure 4*a*) and microvilli penetrating the cuticle at the opposite surface (figure 4*c*,*e*). The apical end of the cells contains secretory vesicles (figure 4*d*) and cilia (figure 5*d*,*e*). Somata of glial and neuronal cells are located in the apical zone of the epidermis (figure 5*c*).

A comparable situation is found in the enteropneust *B. misakiensis*. This species also exhibits an intraepidermal nervous system (figure 2*e*,*f*). Antibody staining of SCO-spondin reveals numerous radially arranged structures in the posterior end of the proboscis in early juveniles. These structures span the entire epidermis in the baso-apical direction (figure 1*a*). Notably, the SCO-K10-LIR is restricted to the posteriodorsal part of the proboscis, where the intraepidermal proboscis plexus is located. Observations using TEM show that the somata of the cells mostly reside in the apical section of the epidermis (figure 5*g*,*h*). The cells also contain prominent intermediate filaments and funnel-shaped endfeet abutting the basal lamina at the inner surface, as well as distinct apical microvilli and cilia at the apical cell surface (figures 4*f*,*g* and 5*h*,*i*). This result reveals, for the first time, the existence of radial glial cells—and glial cells in general—in Hemichordata [9]. The intraepidermal nervous system of the echinoderm *A. gibbosa* is also characterized by prominent radially arranged cells traversing the entire intraepidermal nerve cord and the adjacent epidermis in adult specimens (figure 2*c*,*d*). In agreement with a previous study [29], immunohistochemical analysis unveils distinct SCO-K10-LIR displayed by these cells in *A. rubens* (figure 1*b*). Ultrastructural investigation of the nerve cord in both asteroidean echinoderm species shows the presence of dense bundles of intermediate filaments within these cells (figures 4*i* and 5*k*,*l*). Further on, these cells traverse the entire epithelium with their somata mostly located in the apical neuroepithelium (figure 5*k*,*l*), and bear prominent funnel-shaped basal endfeet at the basal lamina (figure 4*i*), as well as apical microvilli and cilia at the apical cell surface (figures 4*j* and 5*m*,*n*). This is in accordance with earlier findings [41]. Using the same antibody, evidence for the presence of radial glial cells has also been reported for the nervous system of holothurian echinoderms [32,42].

Literature screening furthermore revealed the presence of cells (called ‘tanycytes’) with radial glia-like alignment and ultrastructure in the ecdysozoan Scalidophora (Priapulida, Loricifera, Kinorhyncha; see also [43] and figs 1 and 3*b* in [44] for further ultrastructural details). This prompted us to study the intraepidermal nervous system of *P. caudatus* (Priapulida) (figure 2*g*,*h*). Indeed, we found radially aligned SCO-K10-LIR in cells that span the thickness of the intraepidermal nerve cord and the adjacent epidermis in larvae of *P. caudatus* (figure 1*d*).

The ultrastructures of the intraepidermal nervous system of all studied protostomes and echinoderms resemble each other in detail. As the central nervous system of vertebrates ontogenetically originates from epithelia [45], the resemblance of the intraepidermal nervous systems of echinoderms, hemichordates and protostomes provides further support for the homology of epithelial nervous systems in Bilateria, which probably represents the plesiomorphic condition. Within this intraepidermal nervous system, radial glia represent an important non-excitable structural and functional component, and are therefore proposed to be a key feature of all epithelial nervous systems.

Combining all evidence, including the shared topological position and similar ultrastructural composition, the SCO-spondin -LIR, the expression of the *Ofu-SCO* mRNA and the early developmental occurrence, all data strongly support the conclusion that the cells we located in oweniid annelids, as well as in enteropneusts, echinoderms and priapulids, are homologous to the radial glial cells described in chordates. This hypothesis of homology is based on positional and structural homology criteria [46]. Using a comparative approach [36], this implies the evolution of this cell type in the last common ancestor of Nephrozoa (Protostomia and Deuterostomia).

Noteworthily, radial glial cells are absent in the subepidermal ganglia (*sensu* Richter *et al*. [35]) of the protostomian CNS. Although this difference might suggest a convergent evolution of glial cell types, from different origins, in the Protostomia and Deuterostomia, one should keep in mind that radial glia are missing in the ganglia of the peripheral nervous system of vertebrates as well. This is simply because their existence requires both a basal and an apical cell surface, which are present only in an epithelium [12].

Several studies across all major chordate and echinoderm taxa showed that radial glia are secretory [32,38,42,47,48]. In our study, we provided evidence for a secretory protein (SCO-spondin) expressed in all species studied. So far, secretion of SCO-spondin, which is the main component of the so-called Reissner's substance, is best characterized in the vertebrate cerebrospinal fluid. Here, it condenses and forms the main component of a structure termed Reissner's fibre [49], which is present in the central canal of the chordate dorsal nerve cord [50]. Notably, Reissner's fibre is secreted only by a specialized type of glial cells, the radial glia [29,47]. The function of Reissner's fibre has been suggested to participate in the regulation, circulation and production of cerebral spinal fluid of vertebrates [51–53]. Reissner's fibre is also known from tunicates and cephalochordates [54], and it has recently been shown that vertebrate SCO-spondins have indeed orthologues in invertebrate taxa [55]. However, no Reissner's fibre-like structure has been reported outside Chordata. Interestingly, Arendt *et al*. [56] recently hypothesized two major morphological innovations that contributed to set the stage for the evolution of the animal nervous systems: (i) the establishment of a mucociliary sole enabling extracellular digestion in gastric cavities followed by the evolution of a nerve-net, and (ii) folding of the inner surface of these animals into metameric series of gastric pouches, which optimized nutrient provision, thereby allowing larger body plans, subsequent evolution of bilateral symmetry and the evolution of specialized nervous subsystems. Strikingly, in this context, Reissner's fibre is interpreted as a possible remnant of a mucociliary sole. Our investigations support a secretion of the Reissner's fibre component SCO-spondin in several non-chordate taxa. Thus, the secretory character of radial glial cells appears to be maintained throughout their various evolutionary adaptations, providing a mucociliary sole, components of the cuticle or Reissner's fibre.

In vertebrates, it is well known that radial glial cells have a major impact on early neuronal development. So far, neuro- and gliogenesis originating from radial glial cells are only described for chordates and, partially, for echinoderms [13–15,17–19,57]. However, as our comparative investigation suggests the presence of radial glial cells in the last common ancestor of Nephrozoa, it is tempting to speculate about a possible homology of a neuronal stem cell system across nephrozoan taxa in general. Future studies focusing on neuro- and gliogenesis in protostomes with secretory radial glial cells will help to elucidate this hypothesis.

## Supplementary Material

Table S1. Sampling sites and fixation of specimens.; Figure S2. Comparison of conserved protein domains of Ofu-SCO with that of other members of the Thrombospondin-family.

## Data accessibility

The figures and table supporting this article have been uploaded as the electronic supplementary material.
