# Supplementary material for: Early evolution of radial glial cells in Bilateria
Source: Proc Biol Sci. 2017 Jul 19;284(1859):20170743. doi: 10.1098/rspb.2017.0743 (PMC5543218; doi:10.1098/rspb.2017.0743)
Supplement: Table S1. Sampling sites and fixation of specimens.; Figure S2. Comparison of conserved protein domains of Ofu-SCO with that of other members of the Thrombospondin-family. [file rspb20170743supp1.pdf]

Early evolution of radial glial cells in Bilateria

Authors

Conrad Helm[a,1,*], Anett Karl[b,c,d,1], Patrick Beckers[e], Sabrina Kaul-Strehlow[f], Elke Ulbricht[g], Ioannis Kourtesis[a], Heidrun Kuhrt[b], Harald Hausen[a], Thomas Bartolomaeus[e], Andreas Reichenbach[b] and Christoph Bleidorn[h,*]

[a] Sars International Center for Marine Molecular Biology, University of Bergen, 5008 Bergen, Norway

[b] Paul-Flechsig-Institute for Brain Research, University of Leipzig, 04103 Leipzig, Germany

[c] Translational Center for Regenerative Medicine, University of Leipzig, 04103 Leipzig, Germany

[d] Carl-Ludwig-Institute for Physiology, University of Leipzig, 04103 Leipzig, Germany

[e] Institute of Evolutionary Biology and Ecology, University of Bonn, 53121 Bonn, Germany

[f] Department of Molecular Evolution and Development, University of Vienna, 1090 Vienna, Austria

[g] Biotechnology Center, Technische Universität Dresden, 01307 Dresden, Germany

[h] Museo Nacional de Ciencias Naturales, Spanish National Research Council (CSIC), 28006 Madrid, Spain

[1] these authors contributed equally to this work

* Corresponding authors:

Conrad Helm, Sars International Centre for

Marine Molecular Biology, Thormøhlensgt. 55, N-5008 Bergen, Norway, +47 55 58 43 03, conrad.helm@uib.no

Christoph Bleidorn, Museo Nacional de Ciencias Naturales, Department of Biodiversity and Evolutionary Biology, José Gutiérrez Abascal 2, Madrid 28006, Spain, +34 914111328 ext. 1182, christoph.bleidorn@gmail.com

Table S1. Sampling sites and fixation of specimens.

**Species**	***Owenia fusiformis***<br><br>Delle Chiaje, 1844 (Oweniidae, Annelida)	***Balanoglossus misakiensis***<br>(Kuwano, 1902) (Ptychoderidae, Enteropneusta)	***Asterias rubens***<br>Linnaeus, 1758 (Asteriidae, Echinodermata)	***Priapulus caudatus***<br><br>Théel, 1906 (Priapulidae, Priapulida)
**Age of observed specimens and sampling site**	adult;<br>Saint-Efflam, Brittany, France	17 days post settlement; adults were collected in Aomori, Asamushi, Japan (see [S1] for larval rearing)	adult; Helgoland, Germany	lorica larvae were used; adults originate from Gullmarsfjord, Fiskebäksil, Sweden (see [S2] for larval rearing)
**Fixation (TEM)**	2 % paraform-aldehyde in phosphate buffered saline + 2.5 % glutaraldehyde, overnight at 4°C	2.5% glutaraldehyde with ruthenium red in PBS (0.05 M PB + 0.3 M NaCl) for 30 min on ice; postfixed in 2% OsO_4 for 45 min	2 % paraformaldehyde in phosphate buffered saline + 2.5 % glutaraldehyde, overnight at 4°C	-
**Fixation (IHC)**	4 % paraform-aldehyde in 0.1 M phosphate buffered saline (pH 7.4), overnight	4 % paraform-aldehyde in 0.1 M phosphate buffered saline (pH 7.4), overnight	4 % paraform-aldehyde in seawater, overnight	4 % paraform-aldehyde in filtered sea water, 1h, RT
**Embedding medium (TEM)**	see methods	for embedding see [S1]	see methods	-

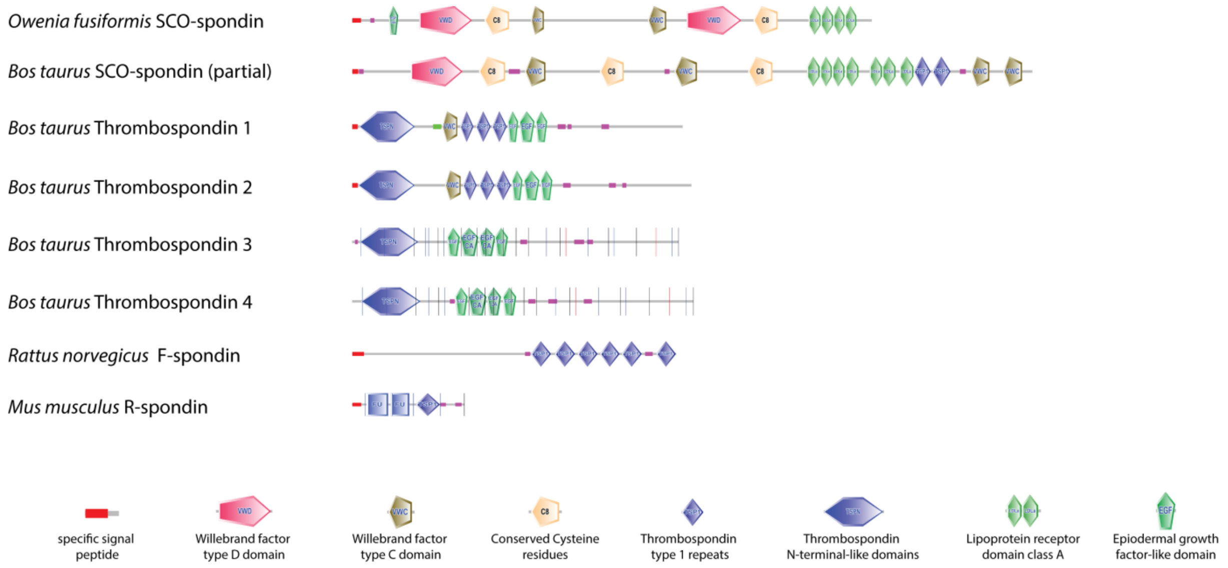

Figure S2. Comparison of conserved protein domains of *Ofu-SCO* with that of other members of the Thrombospondin-family. Main domains are named in the scheme. All protein images are generated using SMART [S3, S4], modified and licensed under a Creative Commons Attribution-Share Alike 3.0 Unported (creativecommons.org/licenses/by-sa/3.0/legalcode).

Supplemental References:

S1. Kaul-Strehlow, S., Urata, M., Minokawa, T., Stach, T., & Wanninger, A. (2015). Neurogenesis in directly and indirectly developing enteropneusts: of nets and cords. *Org. Divers. Evol.*, *15*, 405-422.

S2. Martín-Durán, J. M., Wolff, G. H., Strausfeld, N. J., & Hejnol, A. (2016). The larval nervous system of the penis worm Priapulus caudatus (Ecdysozoa). *Phil. Trans. R. Soc. B*, *371*, 20150050.

S3. Schultz, J., Milpetz, F., Bork, P., & Ponting, C.P. (1998). SMART, a simple modular architecture research tool: Identification of signaling domains. *PNAS, 95, 5857-5864*.

S4. Letunic, I., Doerks, T., Bork, P. (2014). SMART: recent updates, new developments and status in 2015. Nucleic Acids Res., *doi:10.1093/nar/gku949*.
